# A European study investigating patterns of transition from home care towards institutional dementia care: the protocol of a RightTimePlaceCare study

**DOI:** 10.1186/1471-2458-12-68

**Published:** 2012-01-23

**Authors:** Hilde Verbeek, Gabriele Meyer, Helena Leino-Kilpi, Adelaida Zabalegui, Ingalill Rahm Hallberg, Kai Saks, Maria Eugenia Soto, David Challis, Dirk Sauerland, Jan PH Hamers

**Affiliations:** 1 Department of Health Services Research, CAPHRI School for Public Health and Primary Care, Maastricht University, P.O. Box 616, 6200 MD Maastricht, the Netherlands; 2 School of Nursing Science, Faculty of Health, Witten/Herdecke University, Witten, Germany; 3 Department of Nursing Science, Faculty of Medicine, University of Turku, Turku, Finland; 4 South-West Hospital District of Finland, Turku, Finland; 5 Fundacíó Privada Clinic per la Recerca Biomedica, Hospital Clinic of Barcelona, Barcelona, Spain; 6 Lund University, Lund, Sweden; 7 Department of Internal Medicine, University of Tartu, Tartu, Estonia; 8 Department of Geriatric Medicine, Toulouse University Hospital, Toulouse, France; 9 Personal and Social Services Research Unit, Faculty of Medical and Human Sciences, University of Manchester, Manchester, UK; 10 Faculty of Management and Economics, Witten/Herdecke University, Witten, Germany

**Keywords:** Dementia, Long-term care, Professional home care, Nursing homes

## Abstract

**Background:**

Health care policies in many countries aim to enable people with dementia to live in their own homes as long as possible. However, at some point during the disease the needs of a significant number of people with dementia cannot be appropriately met at home and institutional care is required. Evidence as to best practice strategies enabling people with dementia to live at home as long as possible and also identifying the right time to trigger admission to a long-term nursing care facility is therefore urgently required. The current paper presents the rationale and methods of a study generating primary data for best-practice development in the transition from home towards institutional nursing care for people with dementia and their informal caregivers. The study has two main objectives: 1) investigate country-specific factors influencing institutionalization and 2) investigate the circumstances of people with dementia and their informal caregivers in eight European countries. Additionally, data for economic evaluation purposes are being collected.

**Methods/design:**

This paper describes a prospective study, conducted in eight European countries (Estonia, Finland, France, Germany, Netherlands, Sweden, Spain, United Kingdom). A baseline assessment and follow-up measurement after 3 months will be performed. Two groups of people with dementia and their informal caregivers will be included: 1) newly admitted to institutional long-term nursing care facilities; and 2) receiving professional long-term home care, and being at risk for institutionalization. Data will be collected on outcomes for people with dementia (e.g. quality of life, quality of care), informal caregivers (e.g. caregiver burden, quality of life) and costs (e.g. resource utilization). Statistical analyses consist of descriptive and multivariate regression techniques and cross-country comparisons.

**Discussion:**

The current study, which is part of a large European project 'RightTimePlaceCare', generates primary data on outcomes and costs of long-term nursing care for people with dementia and their informal caregivers, specifically focusing on the transition from home towards institutional care. Together with data collected in three other work packages, knowledge gathered in this study will be used to inform and empower patients, professionals, policy and related decision makers to manage and improve health and social dementia care services.

## Background

Action is urgently required to prepare health care services in delivering more cost effective and higher quality care for people with dementia and their informal caregivers. With ageing populations the number of with dementia is ever increasing with no sign yet of a cure for the disease. Symptoms of dementia include a general loss of cognitive, functional and mental capabilities, resulting in diverse needs. Some needs require health care and some are more appropriately met by social care, although the boundaries between these needs are hard to delineate [1]. Demands for health, social and nursing care arise when needs of people with dementia and their caregivers are not fulfilled, usually due to insufficiencies in resources especially related to manage everyday activities and a lack of social network [2].

A common policy principle in European countries nowadays is to enhance resources for home- and community-based care services. This is to enable people with dementia to remain in their own homes for as long as possible, trying to delay institutionalization [1,3-5]. However, it is questionable whether this is an beneficial policy for all people with dementia and their caregivers. The underlying belief is that most older people, including those with complex care needs such as dementia, prefer to live at home since this is a familiar environment [6]. Amongst expected benefits are that people with dementia remain able to maintain their social networks and enjoy a better quality of life [7,8]. Cultural aspects, such as beliefs that children are responsible for older adult's care, could also influence the decision to keep people with dementia at home for as long as possible [8]. The decision to move people with dementia from home care to institutional care is a complex one and is influenced by both patient and caregiver characteristics, available resources and care norms.

Evidence to support the timing of this decision is currently lacking, impeding appropriate timing of institutionalization. For example, knowledge of outcomes and relative benefits such as quality of life and quality of care between home and institutional nursing care for people with dementia in various stages of the disease is currently unknown. To assure more appropriate entry to institutional settings, more information on service provision and related outcomes on quality of life and quality of care is therefore urgently needed.

Cost information is also important for policy makers. It would be dangerous to assume that a shifting balance from institutional towards community care will necessarily be cost reducing [9]. One consequence of this shifting balance is that older people are admitted to care homes when already quite dependent, at later stages of dementia. This can leave families carrying a high burden of care. Furthermore, a recent study suggests that savings on an aggregated level may be variable at the individual level [10]. Policy makers need to understand who pays and benefits from certain interventions, taking account of costs associated with patient location, disease characteristics (e.g. dementia severity) and type of care (informal/formal) [10,11].

Currently, there is little evidence to assist decision making as to when home care or institutional care is more favorable for people with dementia and their families. Similarly, little is known about specific characteristics of people with dementia who benefit most from institutional as opposed to home-and community-based research [6]. Therefore, it is unclear whether preventing admission to an institutional long-term nursing care facility is the best approach for all people with dementia and their informal caregivers. Information on best practice strategies to enable people with dementia with dementia to live at home as long as possible but also to define the right time to trigger the admission to an institutional long-term nursing care facility is therefore urgently required.

Most people with dementia will be admitted to a care facility at some point, since their needs cannot be met appropriately in the home situation [12]. Although many studies have investigated predictors of institutionalization [7,13], it remains unclear if predictors are country specific. Both patient (e.g. severity of cognitive and functional disability) and caregiver characteristics (e.g. perceived burden, coping strategies) could play an important role. There is evidence that rates of institutionalization and time to admission to a nursing home may vary substantially among countries [14-16]. For example, a recent study found that people with dementia with similar characteristics and treatment patterns had a lower risk of nursing home placement in the UK compared with northern Scandinavian countries [14]. Variations in health care structure, dementia care policy, availability of services, cultural values and funding systems may all contribute to these differences. However, there is little primary data across countries to understand variations in predictors of institutionalization for people with dementia.

### Aims and objectives

**Figure 1:**
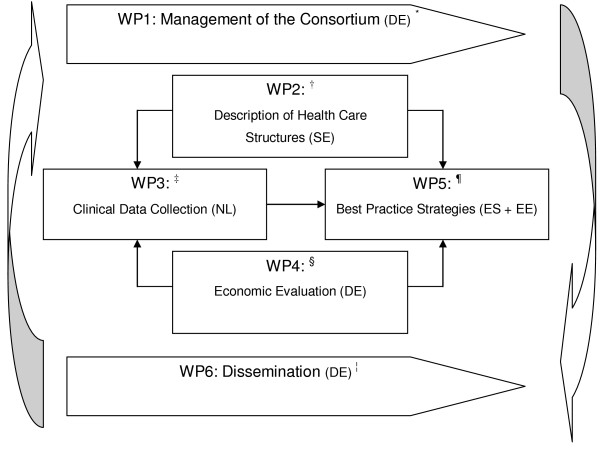
**Overview the European project 'RightTimePlaceCare**. * Work Package (WP) leaders are mentioned in brackets; DE = Germany; SE = Sweden; NL = Netherlands; ES = Spain; EE = Estonia; ^† ^WP2 aims to describe and analyze European health, social and welfare structures and explores intersectorial communication; ^‡ ^WP3 is described in the current study protocol; ^§ ^WP4 aims to analyze cost-benefit ratio of services for relevant stakeholders; WP5 aims to develop best-practice strategies and to deliver meaningful and feasible recommendations for future dementia care; ^¦^. WP6 aims to develop and apply dissemination and implementation strategies of the RTPC project.

This study is part of a large European research project called 'RightTimePlaceCare' (RTPC), which consists of six work packages (WPs) (see Figure 1). The RTPC project aims to improve health services for European citizens with dementia and is explained elsewhere in detail [17]. The current study generates primary data in the transition from professional home care towards institutional nursing care for people with dementia and their informal caregivers for best-practice development. It constitutes the third WP of the RTPC study and has two main objectives:

1) To assess the factors influencing the institutionalization of people with dementia at the time of admission to institutional long-term nursing care facility.

2) To investigate the circumstances and living conditions of people with dementia receiving long-term professional home care or institutional nursing care and their informal caregivers. Emphasis is placed on:

a) Quality of care and quality of life of people with dementia in institutional long-term care and home care;

b) Caregiver burden and quality of life of informal caregivers of people with dementia in institutional long-term care and home care.

In addition, information on direct and indirect costs are collected.

## Methods

### Design

This is a prospective cohort study, conducted in eight European countries (Estonia, Finland, France, Germany, Netherlands, Sweden, Spain, United Kingdom). We attempted to include countries from all over Europe (i.e. northern, southern, eastern and western parts of Europe). A baseline assessment is performed between November 2010 and December 2011 and follow-ups are conducted after 3 months (see Table 1).

**Table 1:** Study design

**Subjects**	**Informed Consent**	**Baseline**	**Follow up**
	(day -14 to -1)	(day 0)	(day 80 to 100)
	
**Group 1** 800 People with dementia (PwD) newly admitted to a facility and their informal caregivers (IC) or next of kin (100 PwD + 100 IC per group per country)	Informed Consent	Baseline assessment	Follow up assessment
	
**Group 2** 1400 PwD receiving professional home care and their IC or next of kin (150 PwD + 150 IC per group per country)	Informed Consent	Baseline assessment	Follow up assessment

### Setting and participants

• The current study focuses on long-term care and consists of two strata:

• Group 1: People with dementia newly admitted to institutional nursing care facilities (i.e. within one to three months after admission) and their informal caregivers or next of kin;

• Group 2: People with dementia who receive professional home care and are at risk of institutionalization (i.e. on the margins of long term care admission) and their informal caregivers or next of kin.

### Setting

A minimum of three different long-term nursing care facilities and three professional home care organizations will be recruited per country, in order to achieve some variation in the sample for recruitment of subjects. To target similar populations across countries with varying health and social care structures in long-term care, we used the following care definitions:

#### Formal long-term care

A range of services for people who need assistance on a continuing basis due to chronic impairments (resulting from physical or mental disability) and a reduced degree of independence and activities of daily living. This central personal care component is frequently provided in conjunction with help in basic medical services such as wound dressing, pain management, medication, health monitoring, prevention rehabilitation or services for palliative care [4]. This care is provided by formal caregivers, professional caregivers that are paid for their job. As such the caregiver may well possess formal professional education, either in health care/nursing and/or social care.

#### Institutional long-term nursing care facilities

Nursing and personal care provided in an institution which at the same time serves as a residence of the care recipient. This should be distinguished from short-term care provided received in institutions, such as respite care and rehabilitation. An institution is a place of collective living where care and accommodation are provided as a package by a public agency, non-profit or private company. Residents may or may not be charged separately for care services and accommodation. In institutional nursing care, a significant part of the the care provided is a mix of health and social services with the health services being largely at the level of nursing services [4,18].

#### Professional long-term home nursing care

Refers to long-term care services that can be provided to patients at home by professional home-nursing organizations and home-help services. This could also include care provided at day-care [4,19]. Examples of home nursing: helping patients with basis needs in activities of daily living, hygiene and other personal care, routine technical nursing procedures, patient education and counseling, psychosocial activities.

### Participants

The study population consists of dyads of people with dementia older than 65 years of age and their informal caregivers, who require formal care/help either from:

1) institutional nursing care facilities. Target n in group one is 800 dyads in total (i.e. 100 per country). We expect a drop-out rate of 15%. Therefore, we aim to include 115 dyads per country in this setting.

2) professional home care organizations. Target n in group two is 1200 (i.e. 150 per country), since we assume that the variance in this group is higher. With an expected drop-out rate of 15%, we aim to include 175 dyads per country in this setting.

#### People with Dementia

Inclusion criteria for people with dementia consist of 1) a formal diagnosis of dementia as diagnosed by an expert assessment (i.e. physician, psychiatrist, neurologist, geriatrician or general practitioner depending on countries' specific diagnostic procedures) and recorded in the medical record; 2) an MMSE score of 24 or below and 3) the presence of an informal caregiver who visits at least twice a month. The severity and type of dementia may vary but will be recorded if information is available. In addition, each group has specific inclusion criteria. People with dementia who are newly admitted to a long-term institutional nursing care facility (Group 1) are included if they live at least 1 month in the institution and no longer than 3 months. People with dementia institutionalized only for a limited period of time a priori (e.g. rehabilitation, respite care) with the intention of moving back home are excluded. People with dementia who receive professional home nursing care (Group 2) should be at risk for institutionalization. This means that a formal caregiver (e.g. registered nurse, general practitioner) judges institutionalization as probable within 3 to 6 months. Reasons for being adjudged at-risk may vary across countries.

#### Informal caregivers

Informal care is defined as care provided by informal caregivers, such as spouses/partners, other members of the household, relatives, friends, neighbors or others, usually but not necessarily with an already existing social relationship to the person they provide care [4]. Caregivers who provide care on a voluntary basis through an organization (such as a church group), or those who provide care as a career are not defined as an informal caregiver.

All main caregivers providing informal care for older people with dementia participating in this study are eligible. The number is limited to one main informal caregiver per older person with dementia, defined as the person who is most involved in care for the people with dementia. For people in institutional nursing care, the term informal caregiver may not be completely appropriate. In this case, the next of kin/significant other is included, being the person who is close to the person with dementia (spouse, children, grand children, other relatives or friends) and most involved in the decisions about their care.

### Measures

Table 2 summarizes all outcome measures. Variables regarding people with dementia and their informal caregivers are chosen based on recent models predicting care demands [2], predicting institutionalization for people with dementia [7,13], and quality of care [20]. Measurement instruments are selected based on their psychometric properties (validity, reliability), clinical utility and appropriateness for the target settings and population. If necessary, questionnaires are translated according to a standardized backward forward translation procedure [21]. Prior to the study, permission was obtained for use and translation of questionnaires.

**Table 2:** Measurement instruments

Variable	Measure	No of items (range*)	Assessment
***People with dementia***			

Socio-demographics	Dataheet	n/a	IC and MR

Comorbidity	Charlson index	n/a	MR

Cognition	MMSE	20 (0-30)	People with dementia

Behavior	NPI-Q	12 (0-36)	Proxy^†^

ADL	KATZ	6 (0-6)	Proxy

Personal and social resources	RUD	n/a	Proxy

Quality of care indicators			Proxy

-nutritional status	Item on weight loss	1 (yes/no)	Proxy

-physical restraints	8 items from MAQ	n/a	Proxy

-pain	MDS based indicators. Presence, frequency, intensity and location.	n/a	Proxy

-pressure ulcer	Presence, intensity	n/a	Proxy

-mortality rate	Register mortality	n/a	Proxy

-mood disturbances/depression	CSDD	19 (0-38)	Proxy

-falls	Frequency and injuries recent falls	n/a	Proxy

Quality of Life	QoL-AD^‡^	13 (13-52)	Self-and Proxy

***Informal caregivers***			

Positive and negative aspects of caregiving	CRA	24 (5 dimensions)	IC

Caregivers burden	ZBI	22 (0-88)	IC

Availability of resources	RUD	n/a	IC

Psychological well-being	GHQ-12	12 (0-12)	IC

Quality of Life informal caregivers	EQ-5D	5 (n/a)	IC

Experiences on QoC	9 items of the CLINT	n/a	IC

	open ended questions	n/a	IC

***Economic evaluation***			

Resource use	RUD	n/a	IC and Proxy

* the underlined score represents the most favorable score; ^† ^at home: informal caregiver; institutional nursing care: formal caregiver; ^‡ ^If a PwD total score is less than 3 on the MMSE, Qol--AD will be assessed only using proxy reports

### Measures for people with dementia

Outcome measures for people with dementia include cognitive status (SMMSE) [22,23], independence in activities of daily living (KATZ) [24], neuropsychiatric symptoms (NPI-Q) [25,26], quality of life (QoL-AD, both self-and proxy assessment) [27], comorbidity (Charlson Comorbidity Index) [28] and medication use. Furthermore, several quality of care indicators as based on recent literature [20] are assessed: nutritional status (one item question 'did the patient experience a weight loss of 4% or more of his weight in the past year?') [29]; falls (falls and fall-related injuries during preceding 3 months), pressure ulcers (presence and severity); depressive symptoms (CSDD) [30]; use of physical restraints (8 items from MAQ) [31]; pain (items from RAI-MDS on presence, frequency and location) [32] and mortality registration. Sociodemographic information includes age, gender, education, marital status, living situation, income, cultural background (i.e. native country, religion, ethnicity) and dementia diagnosis related information.

### Measures for informal caregivers

Outcome measures for informal caregivers include quality of life (EQ-5D) [33]; caregiver burden (ZBI) [34], psychological well-being (GHQ-12) [35]; positive and negative consequences of caregiving (CRA) [36]; experiences on quality of care (9 items from CLINT) [37] and an additional item 'overall, I am satisfied with the quality of care provided by the organization' as rated on a 5-point Likert scale ranging from totally agree to totally disagree); use of personal and social resources and service use (RUD) [38]. Furthermore, an open-ended question is addressed to gain insight in the transition process (either 'what is the main reason for institutionalization?' or 'under what circumstances do you consider institutionalization necessary?'). Sociodemographic variables include age, gender, educational background, marital status, health status, number of visits per month, living situation and relation to people with dementia.

### Costs

Costs are measured using the RUD [38]. It assesses information on resources used (e.g. frequency and duration of hospitalization, visits to health care professionals and type of care, medication use, use of social services) for both patients and informal caregivers. In addition, the RUD investigates caregiver time, defined as time spent on providing basic activities of daily living (e.g. washing, dressing), instrumental activities of daily living (i.e. cooking, handling financial affairs) and time spent on supervision (e.g. preventing self-harm). It also assesses caregiver work status and whether informal caregiving substitutes for paid work. The RUD-FOCA is used to record direct care time in institutional nursing care settings [39].

### Procedures

Trained interviewers collect all data during face-to-face interviews. All interviewers are professionals in health or social care or medical/nursing/social care students with practical experience and at least a Bachelors degree. Furthermore, they received an additional training on the project, all procedures, content of the assessments and completion of questionnaires.

In order to standardize and facilitate data collection, the WP3 leading centre (Maastricht University, the Netherlands) has prepared a manual as a standardized operating procedure (SOP). This manual has three parts: 1) preparation for the interviews, with information on selection of institutions and participants, instructions for interviewers and the study pilot; 2) the interview content, explaining the measurement assessments used during the interviews; and 3) data handling, describing procedures regarding handling and storage of data, data audit and data entry. The manual and all questionnaires were prepared in English and translated to the national languages following a strict instruction to facilitate a standardized layout for data entry purposes.

An instruction meeting took place in September 2010, before the start of recruitment when the manual and procedures were explained by the WP3 team to all main investigators in each country. These main investigators are responsible for transfer of all standardized instructions towards interviewers in each country. At this meeting the inclusion and selection criteria for study participation were discussed, based on a template prepared by the WP3 leading centre and completed by each individual country. Two follow-up meetings were scheduled (February and September 2011) for all researchers to discuss experiences regarding recruitment and data collection, taking country-specific issues into account.

Data are centrally managed by the RTPC coordinating centre (Witten University, Germany) in close contact to the WP3 team who has prepared the SPSS database. Countries prepare either 1) copies of the original data collection forms for keeping at their institutions or 2) scan the original data collection forms for generation of pdf files (or another image file). Completeness and correctness of each file will be checked immediately after scanning by countries. Each country will deliver either 1) the original client record files or 2) a data storage medium comprising the scanned files to the RTPC coordinating centre by secured post or express courier or even personally by a researcher. Data will be delivered twice during the project: after baseline and follow-up assessment. Data entry will be performed centrally at the RTPC coordinating centre. All files will be processed with FormPro Software. Furthermore, the RTPC coordinating centre has prepared a text file database for all open-ended questions (in English), including medication classification according to the ATC coding system, to ensure standardization across countries and enhance data quality.

### Data audit

An external audit of data plausibility and data management will be performed in each country to ensure quality of data collection. The RTPC coordinator (GM) has developed a SOP and data audit checklist and provided training for each independent external auditor. The audit is performed in all countries by a trained external data monitoring auditor, who has a minimum qualification level of a Bachelor's degree in nursing science or a related field of study, is not involved in the study and has good English language skills. The data check covers at least 20% of randomly selected client record files. Names of participating patients and residents remain concealed for the auditor. Furthermore, the auditor will visit at least one or two participating institutional nursing care facilities and one or two participating home care organizations to verify their existence and contribution to participant recruitment.

### Ethical considerations

The Good Epidemiological Practice guidelines recommended by the International Epidemiological Association European Federation are followed. Furthermore, each country has obtained ethical approval from a country specific legal authority for research on human beings (for example a ethical committee specialized in medical or nursing science) to conduct the study in accordance with the national standards and regulations in participating countries. The specific names of each committee are as follows (with reference numbers if appropriate in brackets): Ethics Review Committee on Human Research of the University of Tartu (196/T-3), Ethical Committee of the South-West Hospital District Finland (8/2010), Comite de Protection ds Personnes Sud-Ouest and Outre-Mer Toulouse (09 202 07), Nursing Science Ethical Committee University of Witten/Herdecke, Medical Ethical Committee of the Academic Hospital Maastricht/Maastricht University (MEC 10-5-044), Ethical Committee of the Hospital Clinic Barcelona (2010/6031), Ethical Committee Lund University (20120/538), National Research Ethics Service, North West 5 Research Ethics Committee (11/NW/0003). Prior to data collection, informed consent will be obtained for all participants.

People with dementia and their informal caregivers participate on a voluntary basis and their informed consent is given by their (legal) representatives and if possible by people with dementia themselves. A representative refers to either a legal authorized representative or, if not available, the informal caregiver of the people with dementia who has the power to consent, according to country specific guidelines and regulations. People with dementia who are not able to sign informed consent are asked to assent [40]. Assent is defined as willingness to participate even without full understanding of the complexity and the whole aims of the study. During interviews, a sense of comfort for the participants, with active monitoring of willingness to participate and signs of (non)verbal dissent or distress [40] are provided by the interviewers. Finally, a specific SOP on ethical issues encountered during data collection was developed by the RTPC coordinator. Each researcher is trained to follow these guidelines.

### Statistical analysis

The main objectives of the current study are to investigate factors influencing institutionalization and to explore circumstances and living conditions of people with dementia and their informal caregivers receiving home or institutional nursing care. As this is a cohort study with two strata, the statistical analyses will be primarily a descriptive comparison between the two strata. First, descriptive analyses will be conducted at the level of setting and country at baseline and follow-up. Outcome measures at baseline, follow-up and the changes between baseline and follow-up of the settings as well as between the settings will be described for all countries and each country separately. Therefore, crosstabulations will be used for discrete variables and boxplots, mean/medians and quartiles for continuous variables. Bivariate and correlation analyses will be conducted to relate (socio)demographic variables and outcome measures for People with dementia and informal caregivers. Multivariate regression analyses are conducted per time point and longitudinally. To answer the first objective (factors influencing institutionalization), additional prospective regression analyses will be conducted for the subgroup of participants that were institutionalized during the study period (i.e. baseline assessment in home care (Group 2), follow-up in institutional care (Group 1)). A biostatistician is consulted during preparation of the statistical plan.

### Interpretation of findings

The current study generates primary data on outcomes and costs of long-term nursing care for People with dementia and their informal caregivers, specifically focusing on the transition process of professional formal home care towards institutional care. To interpret these findings, data will be combined with knowledge gathered in the other Work Packages (WPs) of the RTPC study. WP2 (leading centre Lund University, Sweden) analyses European health care structures, social care and welfare systems, advocacy and informal caregiver support systems for patients/consumers with dementia and intersectorial communication covering the continuum of care from informal care, contribution from the civil society, public home care and the intermediate forms of care to the long-term institutional care, including end of life care. WP4 (leading centre Witten/Herdecke University, Germany) assesses costs in long-term dementia care for the time period just before and just after the admission to institutional long-term nursing care facilities from a societal perspective. This means that all relevant costs (direct costs, indirect costs and opportunity costs) will be assessed. Finally, WP5 (leading centres Hospital Clinic of Barcelona, Spain and University of Tartu, Estonia) aims to generate best practice strategies that can be integrated into existing European health and social care systems in order to enable national decision makers to base their decisions on the best knowledge available when they reform the organisation of dementia care. To accomplish this goal, results from WP2 (health care structures), WP3 (primary data collection) and WP4 (economic evaluation) will be integrated with a literature review and Delphi consensus methodology, employing a balance of care methodology, unique in its international configuration [6].

### Study progress

All countries have received formal ethical approval for the study. Baseline data collection started in November 2010 and is intended to end in December 2011. Monthly progress reports are provided by each country as an instrument to supervise enrolment of participants. By November 2011, 1757 dyads of people with dementia and their informal caregivers were included in the study (683 in the institutional care setting and 1074 in home care setting). Furthermore, 996 follow-up interviews had been conducted at that date. Follow-up data collection is expected to end in March 2012. The data audit has been completed by external independent auditors for all eight countries.

## Discussion

The current study is focused on the transition of people with dementia and their informal caregivers from professional home nursing care towards long-term institutional nursing care facilities. This paper describes the research protocol to investigate factors influencing institutionalization and circumstances and living conditions of participants across eight European countries being the third work package in a larger European study called RightTimePlaceCare [17]. Together with data collected in three other work packages, RTPC aims to develop best-practice strategies for need-tailored care while ensuring best available outcomes for people with dementia and their informal care givers at affordable cost-benefit ratios. A RTPC Consortium and Advisory board of expertise were set up, representing nursing, medical, health economics, social care, public policy and other professional disciplines. Several representatives of the project are closely related to national political boards as well as to institutions and political boards of the European Union. This will enable a widespread dissemination of results throughout disciplines, scientific and non-scientific media.

The study is limited by its relatively short follow-up period of 3 months, due to practical restraints. To study the transition process from professional home care towards institutional nursing care, we examine outcomes for two groups of participants who are at the margins of care: 1) people with dementia who have recently been admitted to an institutional nursing care facility and their informal caregivers; 2) people with dementia who are at-risk for institutionalization, receiving professional care at home and their informal caregivers. However, the group of participants who actually switch from home towards institutional care during our study period may be relatively small.

An important strength of the current study is its overall size and the cross-country comparisons. Much variation among care concepts (e.g. what constitutes a nursing home?) and health care structures may exist across European countries, which in turn might affect outcomes and its interpretation. Since this study is part of a larger European project, we were able as one of the first studies to simultaneously collect primary clinical data alongside a thorough analysis of organization of health care structures, execution of an economical evaluation and combine this with recent literature in order to develop best-practice strategies. This knowledge will be used to inform and empower patients, professionals, policy and related decision makers to manage and improve health and social dementia care services

## Abbreviations

ATC: Anatomical Therapeutic Chemical classification system; CLINT: Client interview questionnaire; CRA: Caregiver Reaction Assessment; CSDD: Cornell Scale for Depression in Dementia; EQ5D: EuroQuol, 5 dimensions; GHQ-12: General Health Questionnaire, 12 item version; IC: Informal Caregivers; KATZ: Katz Index of Independence in Activities in Daily Living; MAQ: Maastricht Attitude Questionnaire; MMSE: Mini-Mental State Examination; MR: Medical Record; NPI-Q: Neuropsychiatric Inventory - Questionnaire; PwD: People with Dementia; Quol-AD: Quality of Life in Alzheimer's Disease; RTPC: RightTimePlaceCare; RUD: Resource Utilization in Dementia; SOP: Standard Operating Procedure; WP: Work Package; ZBI: Zarit Burden Interview.

## Competing interests

The authors declare that they have no competing interests.

## Authors' contributions

HV, GM and JH conceived the study protocol and its final design, with participation of HLK, AZ, IRH, KS, MES, DC and DS. DS was responsible for the economical evaluation. HV drafted the manuscript and all authors commented on and approved of the final version of the manuscript.

## Appendix 1

The *RightTimePlaceCare *Consortium partners are:

Coordinator:

Witten/Herdecke University (DE): Gabriele Meyer, professor (scientific coordinator, WP 1 leader), Astrid Schmitz, Anna Renom Guiteras, Dirk Sauerland, professor (WP 4 & 6 leader), Dr Ansgar Wübker, Patrick Bremer.

Consortium Members:

Maastricht University (NL): Jan P.H. Hamers, professor (WP 3 leader); Basema Afram, Hanneke Beerens, Dr Michel H.C. Bleijlevens; Dr Hilde Verbeek; Dr Sandra M.G. Zwakhalen.

Lund University (SE): Ingalill Rahm Hallberg, professor (WP 2 leader); Ulla Melin Emilsson, professor; Dr. Staffan Karlsson.

University of Manchester (UK): David Challis, professor; Caroline Sutcliffe; Dr David Jolley; Anthony Crook;

University of Turku (FI): Helena Leino-Kilpi, professor; Jaana Koskenniemi, Riitta Suhonen, professor; Matti Viitanen, professor; Seija Arve, adj professor; Minna Stolt; Dr. Maija Hupli;

University of Tartu (EE): Kai Saks, professor (WP 5 leader); Ene-Margit Tiit, professor; Jelena Leibur; Katrin Raamat; Angelika Armolik; Teija Tuula Marjatta Toivari;

Fundació Privada Clinic per la Recerca Biomedica, Hospital Clinic of Barcelona (ES): Dr Adelaida Zabalegui (WP 5 leader); Dr Montserrat Navarro; Dr Esther Cabrera (Tecnocampus Mataró).

Gerontôpole, University of Toulouse (FR): Dr Maria Soto; Agathe Milhet; Dr Sandrine Sourdet; Sophie Gillette; Bruno Vellas, professor.

## Pre-publication history

The pre-publication history for this paper can be accessed here:

http://www.biomedcentral.com/1471-2458/12/68/prepub
